# The Effectiveness of Mini‐Pulse Methylprednisolone in the Treatment of Patients With COVID‐19 in the Intensive Care Unit

**DOI:** 10.1002/iid3.70071

**Published:** 2024-11-19

**Authors:** Yousef Mohammadi‐kebar, Shahram Habibzadeh, Saeid Hoseininia, Hasan Ghobadi, Alihossein Samadi, Sousan Mohammadikebar, Ahad Azami, Shahnaz Fouladi, Yousef Amani‐marani, Afshin Habibzadeh

**Affiliations:** ^1^ Department of Internal Medicine, School of Medicine Ardabil University of Medical Sciences Ardabil Iran; ^2^ Department of Infection Diseases, School of Medicine Ardabil University of Medical Sciences Ardabil Iran; ^3^ Department of Anesthesiology and Intensive Care Medicine Ardabil University of Medical Sciences Ardabil Iran

**Keywords:** COVID‐19, intensive care unit, methylprednisolone

## Abstract

**Objective:**

To investigate the effect of mini‐pulse methylprednisolone in the treatment of patients with COVID‐19 hospitalized in the intensive care units (ICU).

**Methods:**

This is a single‐blind parallel non‐randomized clinical trial that will be carried out on 60 hospitalized COVID‐19 patients and conducted between February 2020 and December 2020 in Ardabil City Hospital, Ardabil, Iran. The *t*‐test and chi‐square test were used to compare the results of the two groups. A *p*‐value of less than 0.05 was considered statistically significant. Data were analyzed using Statistical Package for the Social Sciences (SPSS) version 26 software.

**Results:**

The mean (±SD) age of patients was 57.53 ± 13.71 years. Thirty‐five patients (58.3%) were male and 25 (14.7%) were female. Twenty‐eight patients had fever. During admission, the mean (±SD) of the oxygen saturation was 80.73 ± 8.31. No significant relationship was observed between the study variables in the two groups at baseline.

**Conclusion:**

The results of the present study showed that in patients treated with methylprednisolone, blood oxygen saturation increased and heart rate and breathing rate decreased significantly. Also, mini‐pulse treatment with methylprednisolone significantly reduced the number of days of hospitalization and the incidence of mortality.

## 1 Introduction

The new coronavirus, officially named SARS‐CoV‐2, is a beta coronavirus that first broke out in Wuhan, China in December 2019 [1]. The World Health Organization has declared COVID‐19 as the sixth public health emergency [2, 3, 4]. Similar diseases such as Middle East respiratory syndrome coronavirus (MERS‐COV) and SARS‐COV, which have occurred and been studied in previous years, have similarities with COVID‐19, and the necessary treatments used in them have been proposed with the possibility of having an effect on this disease. Several drugs, such as ribavirin, interferon, lopinavir‐ritonavir, and corticosteroids have been used in patients with SARs or MERS, but the effectiveness of some of them is contradictory [5].

Based on the previous information about SARS and MERS diseases, the use of pulse coronet cannot be effective in COVID‐19 patients. However, based on laboratory studies and the findings of some studies, pulse coronet can reduce the severity of lung inflammation and somehow prevent the worsening of the course of the disease [6, 7]. However, in a recent study, it has been stated that the use of low‐dose corticosteroids for a short period of time has been able to improve symptoms and computed tomography (CT) scan findings in COVID‐19 patients [8].

Studies have shown that the rapid clinical deterioration of patients with COVID‐19 is closely related to excessive inflammation (also called cytokine storm). When the cytokine storm mounts, T cells, macrophages, and natural killer cells rapidly proliferate and become hyperactivated, releasing massive inflammatory cytokines that lead to apoptosis of the pulmonary epithelium and endothelial cells, destruction of the pulmonary microvasculature and alveolar cells, and ultimately epithelial cells. It becomes vascular, alveolar edema and hypoxia, and finally acute respiratory distress syndrome (ARDS), which is the main cause of death in patients with COVID‐19 [7, 8]. Therefore, apart from active antiviral therapy, inhibiting the excessive inflammatory response and preventing tissue damage are also the focus of treatment for COVID‐19.

The use of a complete pulse or mini‐pulse should not be used as the preferred treatment in COVID‐19 patients with mild to moderate involvement or severe involvement in the early stages. However, due to the possibility of effectiveness, it can be used as the last shot in controlling the high inflammation of the disease and suppressing the cytokine storm in patients who are intubated and hospitalized in the intensive care unit (ICU) and their condition is getting worse every moment [6]. Therefore, in the present study, we intend to examine the effect of mini‐pulse methylprednisolone in the treatment of patients with COVID‐19 hospitalized in the ICU.

## 2 Material and Methods

### 2.1 Study Design

This is a single‐blind, parallel non‐randomized clinical trial that will be carried out on 60 hospitalized COVID‐19 patients conducted between February 2020 and December 2020 in Imam Khomeini Hospital, Ardabil, Iran. This study was conducted after approval of the Ethics Committee in Biomedical Research, the Vice Chancellor for Research and Technology of Ardabil University of Medical Sciences, and the Iranian Clinical Trial Registration Center (IRCT20170716035126N4) and considering the Helsinki Agreement. The intervention was conducted in full compliance with international guidelines and without any harm to pregnant mothers. All implementation steps were explained to the participants, and it was ensured that all patient information was kept confidential.

### 2.2 Study Community and Eligibility Criteria

Patients aged over 18 years admitted to ICU with moderate to severe COVID‐19 infection need respiratory support, PaO_2_/FiO_2_ less than 300, progression of disease severity, and not responding to standard treatment. Patients with uncontrolled diabetes mellitus, active bleeding, active bacterial or fungal infection, positive procalcitonin, hypersensitivity to corticosteroids, and previous immunosuppressor or corticosteroid use were considered as exclusion criteria.

### 2.3 Study Protocol

The intervention group will receive intravenous mini‐pulse Methylprednisolone 250 mg for 3 days to treat of COVID‐19. The control group will also be selected from among the patients who did not consent to receive mini‐pulse after matching in terms of age and sex. The control group will receive a standard care regimen for COVID‐19. This standard care for COVID treatment is the guidelines and treatment protocols for COVID that have been announced by the Ministry of Health. Clinical findings, vital signs, laboratory tests, and radiological results were recorded in the beginning (baseline) and at the end of the study.

### 2.4 Outcomes

Discharge from the hospital (survival) and death were considered as primary outcomes, and oxygen saturation (SPO_2_), hospitalization length, changes in the vital sign and laboratory values, and clinical findings were considered secondary outcomes which was evaluated in patients in the end of study.

### 2.5 Data Analysis

The mean ± standard deviation (SD) was used to describe the quantitative data, and the frequency and percentage were used for the qualitative data. The Kolmogorov‐Smirnov test was used to assess the normality of the variables. The *t*‐test and chi‐square test were used to compare the results of the two groups. A *p*‐value of less than 0.05 was considered statistically significant. Data were analyzed using Statistical Package for the Social Sciences (SPSS) version 26 software.

## 3 Results

Based on K‐S test, all of the data were normal, and we used parametric tests to analyze the data. The mean (±SD) age of patients were 57.53 ± 13.71 years. Thirty‐five patients (58.3%) were male and 25 (14.7%) were female. Twenty‐eight patients had fever. During admission, the mean (±SD) of the oxygen saturation was 80.73 ± 8.31. No significant relationship was observed between the study variables in the two groups at baseline (Table 1).

**Table 1:** Demographic and clinical findings of patients at the baseline.

Variables		Methylpredisolone (*N* = 30)	Standard care (*N* = 30)	*p* value
Age, Mean (SD)		57.37 (15.56)	57.70 (11.84)	0.926
Male, *n* (%)		18 (60)	17 (56.7)	0.793
Vital Sign, Mean (SD)	Heart Rate HR	91.55 (17.41)	92.53 (15.45)	0.820
	Systolic blood pressure BP	117.28 (27.49)	124.83 (17.93)	0.215
	Diastolic BP	73.72 (9.89)	77.00 (8.57)	0.179
	Respirratory Rate RR	22.73 (5.50)	24.83 (6.25)	0.173
Laboratory characteristics, Mean (SD)	White blood cell WBC	7323.33 (3475.40)	7773.67 (4507.92)	0.666
	Polymorph unclear PMN	87.17 (8.67)	79.87 (11.64)	0.626
	Lymphocyte	14.70 (7.59)	16.30 (11.04)	0.516
	Hemogolobin Hb	13.41 (1.39)	13.50 (1.75)	0.812
	Platelet	196,123.33 (89085.86)	208,866.67 (112,483.48)	0.628
	Aspartate aminotransferase AST	62.03 (34.32)	81.56 (57.08)	0.119
	Alanine transaminase ALT	43.20 (27.52)	44.85 (38.08)	0.851
	Alkaline phosphatase ALP	227.50 (110.55)	194.32(140.17)	0.331
	Ferritin	673.30 (418.63)	66,317 (462.55)	0.931
	Erythrocyte sedimentation ESR	38.67 (20.99)	38.77 (20.99)	0.984
Clinical finding, *N* (%)	Gastrointestinal symptoms	14 (46.7)	20 (66.7)	0.118
	Myalgia	25 (83.3)	29 (96.7)	0.085
	Dyspnea	22 (73.3)	21 (70)	0.774
	Cough	28 (93.3)	30 (100)	0.150
	Fever	16 (53.3)	12 (40)	0.192
	Chest pain	7 (23.3)	10 (33.3)	0.390

No significant relationship was observed between SPO_2_ of both groups at the baseline (*p* = 0.077), and both of them were lower than the normal range. Pulmonary involvement has been found in all patients, and all of them needed to receive oxygen therapy. Bilateral involvement (GGO and consolidation) was found more than unilateral. Most patients (45%) had 50%–70% pulmonary involvement (Table 2).

**Table 2:** Patients' status and pulmonary involvements level at baseline.

variables		Methylprednisolone *N* = 30	Standard care *N* = 30	*p* value
SPO2, Mean (SD)		80.69 (6.81)	80.77 (9.66)	0.077
Type of oxygen therapy, *N* (%)	Reserve mask	22 (73.3)	19 (63.3)	0.405
	Noninvasive ventilation	8 (26.7)	11 (36.7)	
Type of pulmonary involvement, *N* (%)	Unilateral ground‐glass opacity (GGO)	3 (10)	5 (16.7)	0.447
	Bilateral GGO	27 (90)	25 (83.3)	
	Unilateral consolidation	6 (20)	4 (13.3)	0.488
	Bilateral consolidation	24 (80)	26 (86.7)	
Pulmonary involvment percentage, *N* (%)	(50%)	3 (10)	4 (13.3)	0.846
	(50%–70%)	13 (43.3)	14 (46.7)	
	70%	14 (46.7)	12 (40)	

Totally, 22 (36.7%) of patients died due to COVID and its complications. Half of THE standard care group died and had a significant relationship in discharge and death between the two groups (*p* = 0.032). SPO2 increased in both groups, and the methylprednisolone group significantly more than standard care group (88.77 vs. 81.19, *p* < 0.001). Hospitalization length in the methylprednisolone group significantly lower than others. There was no significant difference between clinical findings rate in two groups (Table 3).

**Table 3:** Primary and secondary outcomes in patients in the end of study.

Variables		Methylprednisolone *N* = 30	Standard care *N* = 30	*p* Value
Primery Outcome	Discharge, *N* (%)	23 (76.7)	15 (50)	0.032
	Death, *N* (%)	7 (23.3)	15 (50)	
SPO2, Mean (SD)		88.77 (5.21)	81.19 (7.49)	0.001
Hospitalization length, Mean (SD)		17.60 (8.09)	22.97(10.62)	0.032
Clinical finding, *N* (%)	GI Symptoms	2 (6.7)	4 (13.3)	0.995
	Myalgia	2 (6.7)	3 (10)	
	Dyspnea	5 (16.7)	9 (30)	
	Cough	5 (16.7)	7 (23.3)	
	Fever	0	0	
	Chest pain	1 (3.3)	2 (6.7)	

No patient had fever. There was significant relationship in WBC, Platelets, ALT, AST, ferritin, and ESR between baseline and the end of the study in both groups. Vital signs and laboratory characteristics of the end of study are shown in Table 4.

**Table 4:** Vital sign and laboratory characteristic at the end of study as secondary outcomes in two groups.

Variables		Methylprednisolone *N* = 30)	Standard care *N* = 30)	*p* Value
Vital sign, mean (SD)	HR	81.13 (11.97)	92.27 (11.36)	0.42
	Systolic BP	125.0 (10.99)	126.67 (19.13)	0.91
	Diastolic BP	77.67 (12.23)	77.33 (10.73)	0.831
	RR	19.40 (4.41)	27.27 (7.19)	0.24
Laboratory characteristics, mean (SD)	WBC	11,300.00 (5065.30)	12,651.72 (5683.91)	0.001
	PMN	84.88 (10.77)	81.28 (8.70)	0.637
	Lymphocyte	11.53 (8.48)	13.38 (7.46)	0.263
	Hemoglobin	12.49 (1.50)	12.34 (2.30)	0.92
	Platelet	169,820.00 (108,852.59)	289,103.45 (130,900.27)	0.009
	AST	54.57 (31.98)	128.40 (285.57)	0.001
	ALT	59.73 (51.17)	89.64 (142.84)	0.014
	ALP	195.60 (96.87)	197.82 (88.41)	0.85
	Ferritin	599.37 (280.66)	896.25 (662.62)	0.013
	ESR	34.40 (12.61)	43.31 (32.94)	0.001

## 4 Discussion

This study was designed and implemented with the aim of evaluating the effect of mini‐pulse methylprednisolone in the treatment of patients with COVID‐19 hospitalized in ICU. Our study showed that in patients who were treated with methylprednisolone, blood oxygen saturation increased and heart rate and respiration rate decreased significantly. Also, the average number of days of hospitalization until discharge was shorter and death was lower in the methylprednisolone group. It is reasonable to assume that any effect shown in the trial may be

attributable to methylprednisolone. Currently, the evidence on the therapeutic effects of methylprednisolone in patients with COVID‐19 is somewhat mixed.

In a Spanish clinical trial, after a 3‐day course of treatment with methylprednisolone at a dose of 120 mg/day, respiratory status, mortality, and inflammatory phenotype were not significantly different between the methylprednisolone and placebo groups [9]. In one study, the univariate Cox proportional hazard model showed that the group receiving a 3‐day steroid pulse at a dose of 500 to 1000 mg/day had a reduced 28‐day mortality (hazard ratio 0.30; [95% CI, 0.44–0.20]; *p* < 0.01). After adjusting for intervening variables (age, sex, remdesivir, baricitinib, and favipiravir), using the multivariate Cox proportional hazards model, the group that received steroid pulse for 3 days with a dose of 500–1000 mg/day, mortality 28 experienced fewer days (0.50 [0.30–0.85], *p* = 0.01) [10]. Some studies showed that the use of hydrocortisone is not effective in reducing mortality in patients with COVID‐19 [11, 12].

It seems that the method of treatment, drug dosage, and duration of intervention are the causes of these differences. Differences between the corticosteroids used in these trials with respect to their affinity for the glucocorticoid receptor, intrinsic anti‐inflammatory effect, and mineralocorticoid potency may possibly explain the negative results obtained with hydrocortisone and the positive results obtained with dexamethasone and methylprednisolone [13, 14, 15]. It is noteworthy that these clinical trials in patients with moderate and severe COVID‐19 pneumonia have reported positive results of treatment with pulse methylprednisolone, but without sufficient independent evidence for its effectiveness, because of analysis for confounding variables. It is unregulated, and other factors, such as intubation, may contribute to recovery. In addition, these studies may have a high risk of bias because the methods used to blind the researchers with respect to the intervention are unknown and present a high risk of reporting bias.

Despite initial concerns about their potentially harmful effects in COVID‐19 patients [7]. Corticosteroids were used early in the epidemic due to their beneficial effects in ARDS and the known inflammatory pathophysiology of COVID‐19 [16, 17]. One study reported reduced mortality, ICU admission, and mechanical ventilation rates among patients requiring oxygen therapy treated with methylprednisolone [18]. However, a paradigm shift in favor of corticosteroids occurred with the publication of the preliminary results of the recovery trial showing improved 28‐day survival with dexamethasone (6 mg/day) in hospitalized patients with COVID‐19 [19].

A cytokine storm, characterized by increased levels of cytokines (e.g., IL‐6), can cause or worsen acute respiratory distress syndrome and multiorgan failure, which is a critical issue in COVID‐19 [20]. In patients with a severe form of COVID‐19, the high mortality rate due to the rapid development of pneumonia secondary to COVID‐19 has made the need to treat with high doses of corticosteroids (pulsed) for a longer period of time [21]. Elevated serum levels of IL‐6 and CRP as an inflammatory marker have been shown to be associated with the severity of COVID‐19 and can be used as a predictive factor for disease risk [22]. Although these changes were not significant in our study, it seems that better outcomes are obtained if methylprednisolone pulse therapy is started earlier in the course of the disease.

Several limitations are considered in the present study. First, to further measure the clinical effect of mini‐pulse methylprednisolone, clinical symptoms were not assessed as outcomes. Also, this trial was only conducted on hospitalized adults because in 2019 in Iran, where our research was conducted, most of the patients with COVID‐19 were under 60 years old and especially under 50 years old [23].

Hence, the results may not be generalizable to other populations. Also, due to the large number of patients in our treatment center, long‐term follow‐up was not possible. Also, since viral load tests are only performed by the Iranian government, these tests were not performed in the present study to more accurately assess treatment response. The small sample size of this research is another limitation of the current research, and we have no option to increase the sample. Therefore, future studies with larger sample sizes are necessary to reduce the risk of type II error.

## 5 Conclusion

The results of the present study showed that in patients treated with methylprednisolone, blood oxygen saturation increased and heart rate and breathing rate decreased significantly. Also, mini‐pulse treatment with methylprednisolone significantly reduced the number of days of hospitalization and the incidence of mortality.

## Author Contributions

**Yousef Mohammadi‐kebar** and **Saeed Hoseininia:** conceptualization, writing–original draft. **Hasan Ghobadi, Alihosein Samadi, Sousan Mohammadikebar,** and **Afshin Habibzadeh:** analyzed the data, and reviewed and edited it. **Ahad Azami, Shahnaz Fouladi,** and **Yousef Amani‐marani:** contributed to the questionnaire, review, and editing. All the authors have read and approved the final manuscript.

## Conflicts of Interest

The authors declare no conflict of interest.

## Data Availability

The data supporting the study are available via the authors.
